# Synthetic nanoscale electrostatic particles as growth factor carriers for cartilage repair

**DOI:** 10.1002/btm2.10043

**Published:** 2016-11-18

**Authors:** Nisarg J. Shah, Brett C. Geiger, Mohiuddin A. Quadir, Nasim Hyder, Yamini Krishnan, Alan J. Grodzinsky, Paula T. Hammond

**Affiliations:** ^1^ Dept. of Chemical Engineering Massachusetts Institute of Technology 77 Massachusetts Avenue Cambridge MA 02139; ^2^ David H. Koch Institute for Integrative Cancer Research, Massachusetts Institute of Technology 500 Main Street Cambridge MA 02142; ^3^ Dept. of Biological Engineering Massachusetts Institute of Technology 77 Massachusetts Avenue Cambridge MA 02139; ^4^ Dept. of Mechanical Engineering Massachusetts Institute of Technology 77 Massachusetts Avenue Cambridge MA 02139; ^5^ Dept. of Electrical Engineering and Computer Science Massachusetts Institute of Technology 77 Massachusetts Avenue Cambridge MA 02139; ^6^ Institute for Soldier Nanotechnologies, Massachusetts Institute of Technology 500 Technology Square Cambridge MA 02142; ^7^ Present address: Dept. of Coatings and Polymeric Materials North Dakota State University Fargo ND 58108

**Keywords:** compounds/materials, drug delivery, nanoparticles, regenerative medicine, osteoarthritis

## Abstract

The efficient transport of biological therapeutic materials to target tissues within the body is critical to their efficacy. In cartilage tissue, the lack of blood vessels prevents the entry of systemically administered drugs at therapeutic levels. Within the articulating joint complex, the dense and highly charged extracellular matrix (ECM) hinders the transport of locally administered therapeutic molecules. Consequently, cartilage injury is difficult to treat and frequently results in debilitating osteoarthritis. Here we show a generalizable approach in which the electrostatic assembly of synthetic polypeptides and a protein, insulin‐like growth factor‐1 (IGF‐1), can be used as an early interventional therapy to treat injury to the cartilage. We demonstrated that poly(glutamic acid) and poly(arginine) associated with the IGF‐1 via electrostatic interactions, forming a net charged nanoscale polyelectrolyte complex (nanoplex). We observed that the nanoplex diffused into cartilage plugs in vitro and stimulated ECM production. In vivo, we monitored the transport, retention and therapeutic efficacy of the nanoplex in an established rat model of cartilage injury. A single therapeutic dose, when administered within 48 hr of the injury, conferred protection against cartilage degradation and controlled interleukin‐1 mediated inflammation. IGF‐1 contained in the nanoplex was detected in the joint space for up to 4 weeks following administration and retained bioactivity. The results indicate the potential of this approach as an early intervention therapy following joint injury to delay or even entirely prevent the onset of osteoarthritis.

## 1 Introduction

Osteoarthritis (OA) is a complex degenerative musculoskeletal disease caused by a combination of mechanical and biological factors that result in the destruction of cartilage, which is often irreversible.^1^ Clinically, there is a lack of early intervention, pro‐anabolic therapies that can stem or reverse the progression of the disease.^2^ The vast majority of clinical therapies are administered to provide temporary joint pain relief after the manifestation of the disease and include high molecular weight hyaluronic acid, glucocorticoids, and nonsteroidal anti‐inflammatory drugs (NSAIDs). These therapies have no inherent cartilage regenerative properties. Steroids and NSAIDs may even suppress chondrogenesis if administered in repeated high doses. Since cartilage tissue is avascular, therapeutic agents must be delivered locally to intra‐tissue targets through the extracellular matrix (ECM) via intra‐articular (IA) injections. While local delivery mitigates systemic toxicity, all therapeutic drugs delivered through IA injections are cleared rapidly, with a clearance half‐life that is weakly proportional to the molecular weight and spans from a few minutes to a day.^3^, ^4^ Hence, pain‐relief mediated by the drugs is transient, if it occurs at all, and often insufficiently effective.^5^ This timescale is also unsuitable for resolving inflammation and stimulating repair of cartilage, which can require the sustained presence of large molecules, such as growth factors, at therapeutic levels.

The use of growth factors as therapeutics to reverse or inhibit cartilage degradation has been an important focus in cartilage research. By delivering agents that promote cartilage repair, it may be possible to create a treatment that not only alleviates pain for extended periods, but can also result in full, functional joint recovery. To this effect, the use of pro‐anabolic growth factors has been explored as a means to stimulate cells to increase chondrogenesis via mitogenic activity and to produce cartilage matrix. The roles of the transforming growth factor‐beta^6^ (TGF‐β) growth factor family, insulin‐like growth factor‐1^7^ (IGF‐1), and fibroblast growth factors^7^, ^8^ (FGF‐2/FGF‐18) in signaling chondrocytes to produce cartilage ECM are well known. In our own work, we have examined the pro‐anabolic effects of IGF‐1.^9^, ^10^ Other studies have shown that chondrocytes produce IGF‐1 that can stimulate ECM biosynthesis in native cartilage and tissue engineered constructs in a dose dependent manner.^11^

A key barrier to the clinical use of growth factors for cartilage repair is the delivery of therapeutically relevant amounts of growth factors to target cells over appropriate time scales to induce tissue regeneration.^12^ Although it would be most desirable to deliver growth factors directly to chondrocytes for the greatest and most efficacious results, the diffusion of free growth factors from the joint space to the deep zones of the cartilage is hindered and inefficient.^13^ Transport through cartilage is regulated by tightly packed collagen fibrils and highly charged proteoglycans of the ECM, which account for ∼60–70% and ∼15–35% of the dry weight of the tissue, respectively.^14^ The combination of size and charge limitations makes the cartilage matrix a formidable barrier to the entry of therapeutic molecules and carriers. The direct bolus injection of growth factors results in the rapid clearance of the growth factor out of the joint space before it can be effective as a therapeutic. Thus, supraphysiologic amounts, well above the therapeutically relevant levels, must be introduced to induce a biological effect, which can lead to safety concerns. Encapsulation of growth factors and localized delivery can potentially address many of these issues. However, the incorporation of growth factors into common polymeric drug carriers can lead to exposure to undesirable processing conditions such as temperature and solvent that often denatures the protein. Cytotoxic byproducts may also be created when the polymer itself degrades in confined environments of the body.

We hypothesized that by creating depots of IGF‐1 within the cartilage matrix, IGF‐1 can be presented for long periods of time and provide sustained signaling to promote matrix biosynthesis, chondrogenesis, and protection against cartilage degradation. In this report, we tested this hypothesis by using a growth factor carrier assembled using charge interactions between the IGF‐1 and synthetic polypeptides, negatively charged poly(glutamic acid) (pGlu) and positively charged poly(arginine) (pArg), into a charged nanoscale polyelectrolyte complex (nanoplex). We observed that the IGF‐1 retained bioactivity when incorporated in the nanoplex. In a rodent model for articular cartilage damage, we observed that when the IGF‐1 was delivered from the nanoplex, it remained at a therapeutically relevant level within the joint space over a period of 4 weeks, protected the cartilage from degradation and mitigated joint inflammation by controlling interleukin‐1 (IL‐1) levels following injury.

## 2 Materials and methods

### 2.1 Constructing the nanoplex

Solutions of IGF‐1 (Biovision, Milpitas, CA), poly(l‐glutamic acid) (Alamanda Polymers, Huntsville AL, MW ∼7.5 kDa) and poly(l‐arginine) (Alamanda Polymers, Huntsville, AL MW ∼5.8 kDa) in ultrapure water were prepared. First, the polyGlu was added to the IGF‐1 at a rate of 1 ml/min under high agitation. Next, the polyArg was added to the IGF‐1/polyGlu mixture at a rate of 1 ml/min under high agitation. The final molar concentrations of the components were 1:5:5 (IGF‐1: polyGlu:polyArg) The resulting particles were stirred for 1 hr and passed through 0.22 μm syringe filters. The particles were dialyzed against water using a 10 k MWCO membrane (Spectrum Labs). Particle size and zeta potential were measured using a Zetasizer Nano ZS90 (Malvern).

### 2.2 Cryo‐TEM

Nanoplex samples were prepared and immediately processed for cryo‐TEM imaging. The nanoplex samples were processed using a vitrification robot (Vitrobot, FEI) in which the relative humidity is maintained close to saturation. A 3‐μL drop of the solution was placed on a carbon‐coated lacey substrate supported by a TEM 300 mesh copper grid (Ted Pella). After automatic blotting, the grid was rapidly plunged into liquid ethane at its melting temperature. This resulted in a vitrified film. The vitrified specimen was then transferred under a liquid nitrogen environment to a cryo‐holder (model 626, Gatan Inc., Warrendale, PA) into the electron microscope, Tecnai 20, Sphera (FEI), operating at 200 kV with a nominal underfocus of 2–4 μm. The working temperature was kept below −175°C, and the images were recorded on Gatan 794 MultiScan digital camera and processed with Digital Micrograph 3.6. Particle sizes were quantified using thresholding and particle size counting algorithms within ImageJ (NIH).

### 2.3 BrdU testing for IGF‐1 bioactivity

The ability of IGF‐1 to induce proliferation of an immortalized chondrocyte cell line, CHON‐001 (ATCC) was used to measure bioactivity. 5000 CHON‐001 cells at low passage number (less than P4) were dispensed in each well of a 96 well plate (quantification) in 0.2 ml of growth medium (Dulbecco's Modified Eagle's Medium supplemented with 10% fetal bovine serum and 1% antibiotic solution containing penicillin and streptomycin). Cells were allowed to establish for 24 hr. Nanoplex IGF‐1 and free IGF‐1 at matched concentrations were then added to the wells. Cells were allowed to proliferate for 24, 48, and 72 hr, without media change. At the end of each timepoint, cells were labeled with 10 μM bromodeoxyuridine (BrdU) for another 2 hr (Roche Diagnostics, Mannheim, Germany). Incorporation of BrdU into DNA was detected using chemiluminescence, following the manufacturer's protocol.

### 2.4 Bovine cartilage explant harvest and culture

Three‐millimeter diameter cartilage explants were biopsied from the femoropatellar grooves of young (1–2 weeks old) bovine knee joints (Research 87, Boylston, MA). These explants were trimmed to 1 mm thickness at the superficial zone of the cartilage. Explants were cultured in Dulbecco's Minimal Essential Medium plus 10% v/v fetal bovine serum, supplemented with pen/strep antibiotics, l‐Proline, ascorbic acid, HEPES, sodium pyruvate, and non‐essential amino acids. Explants were cultured for 48 hr after harvest, prior to any experiments to allow the tissue to recover from the harvesting procedure.

### 2.5 ^35^SGAG biosynthesis assay

To assess cartilage matrix biosynthesis in response to treatment, cartilage explants were incubated with IGF‐1 alone, IGF‐1 in nanoplex form, or culture medium in the presence of 35S. Cartilage proteoglycan (such as aggrecan) biosynthesis in this timeframe would incorporate 35S radiolabel into the tissue, which was measured by digesting the tissue with proteinase K and assaying the tissue extract with a liquid scintillation counter (PerkinElmer). Sulfated proteoglycan biosynthesis rate in response to treatment is proportional to the amount of 35S incorporation. Explants were incubated in a media treated with 20 µM IGF‐1, 20 µM IGF‐1 in nanoplex form, or PBS for 24 and 48 hr (*n* = 5 per condition). Each solution was spiked with 5 µCi/ml 35S. At each timepoint, explants were washed 4 times for 15 min in PBS and digested with Proteinase K at 57°C. After digestion, samples were counted by liquid scintillation (PerkinElmer) and converted to a known quantity of sulfate used in biosynthesis.

### 2.6 Penetration of IGF‐1 and PArg into IL‐1 treated cartilage explants

IGF‐1 was labeled with AlexaFluor 647 (ThermoFisher Scientific, Waltham, MA) and PArg was labeled with Rhodamine Red (ThermoFisher Scientific) via NHS ester chemistry for this experiment. Fresh cartilage explants were treated with 10 ng/ml of IL‐1α (R&D Systems) for 4 days to simulate early effects of injury on matrix loss, a treatment that causes ∼20% GAG loss, predominantly from the superficial zone.^15^ After thorough washing to remove phenol red containing media, explants were incubated with labeled IGF‐1 or IGF‐1 nanoplex in PBS for an additional 48 hr. Explants were again washed thoroughly in PBS to remove any weakly bound material. The 3‐mm diameter, 1‐mm thick cylindrical explants were then sectioned along their long axis to yield thin sections 3 mm long and 1 mm wide. These sections were immediately imaged with a Nikon A1R confocal microscope to visualize the diffusion gradient of IGF‐1 or nanoplex IGF‐1 along the 1 mm thickness of the explant.

### 2.7 Animal studies

All animal work performed was approved by the IACUC at MIT as well as the Animal Care and Use Review Office at the U.S. Army Medical Research and Materiel Command. The method to induce OA has been described extensively elsewhere. Briefly, Sprague‐Dawley rats (250–300 g) were used. Surgery was performed on one limb of the animal, whereas the contralateral limb served as the uninjured control. Five animals were used per treatment condition. The patella was dislocated, followed by anterior cruciate ligament (ACL) transection and medial meniscus resection (ACLT + MMx) with micro‐scissors. The joint surface was irrigated and after relocation of the patella, the capsule, and skin (intradermal) were closed. Therapy was administered within 48 hr of surgery and the animals were monitored over time. Under anesthesia, an injection was administered in the intra‐articular space using an insulin‐gauge needle under anesthesia. Similarly, joint aspirates were performed on both joints on a weekly basis after injury, yielding approximately 25 μL of synovial fluid for each aspirate. An enzyme‐linked immunosorbent assay (ELISA) was used to quantify the amount of IL‐1β and IGF‐1 (R&D Systems). For tracking the protein in vivo, IGF‐Alexa Fluor 647 was used. An in vivo imaging system (IVIS) Spectrum preclinical imaging system and Living Image software (Caliper) was used to acquire and quantify the fluorescence in the same animal (ex/em 649/675). Images were taken periodically after injection until the signal was not detectable.

### 2.8 Histology analysis

After euthanasia, the joints were excised, trimmed and fixed in 4% paraformaldehyde (PFA) for 48 hr and transferred to a 70% ethanol solution. Joints were partially decalcified for about 4 hr using a rapid decalcifying formic acid/hydrochloric acid mixture (Decalcifying Solution, VWR). The joint area was cut in cross‐section with a razor blade and embedded in paraffin wax. Sections (5 µm) of the cross section were stained with Safranin‐O and imaged using bright‐field microscopy. Heat‐induced epitope retrieval was used prior to applying immunohistochemical stains for detecting aggrecan and collagen. Anti‐Aggrecan and anti‐collagen antibody (Abcam) was used in a 1:200 dilution in Tris‐buffered saline and with an HRP conjugate for detection.

### 2.9 Statistical analysis

Prism (GraphPad) was used for all analyses. Results are presented as means ± SEM. Data were analyzed by ANOVA and comparisons were performed with a Tukey post hoc test (multiple groups) or a Student's *t* test (two groups). *p* < .05 was considered significant.

## 3 Results

**Figure 1:**
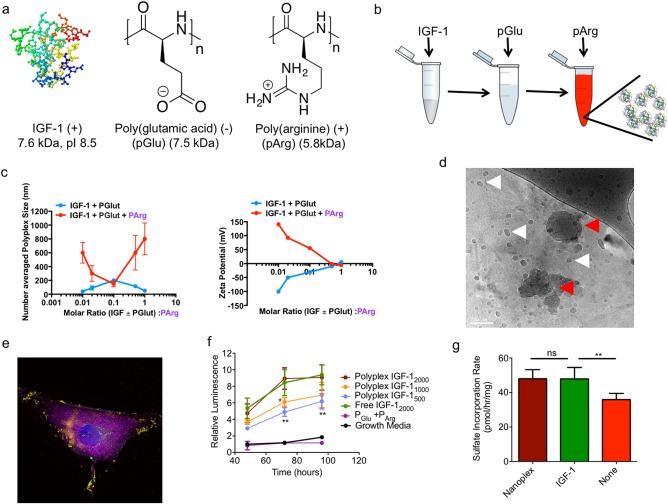
Characterization of IGF‐1 containing nanoplex. (a) Structures and properties of polyelectrolyte materials used in the study (b) Schematic for generating IGF‐1 nanoplex materials. (c) Size and zeta potential as a function of polyelectrolyte molar ratios. (d) cryo‐TEM image of the generated IGF‐1 nanoplex. The white arrows indicate the location of a nanoplex and red arrows indicate nanoplex aggregates (scale bar: 100 nm) (e) Colocalization of the IGF‐1 nanoplex with CHON‐001 cells. The nucleus (blue), membrane (magenta), pArg (red), and IGF‐1 (green) have been stained. Yellow shows assembled nanoplex. (f) Bioactivity of IGF‐1 as measured by BrdU proliferation assay of CHON‐001 cells (*n* = 6 per condition, **p < .01, *p < .05) (g) Rate of cartilage tissue sulfate incorporation in response to treatment with 20 µM nanoplex, 20 µM IGF‐1 alone, or no treatment added to cartilage media consisting of DMEM, 10% FBS, non‐essential amino acids, pyruvate, HEPES, ascorbate, and proline (*n* = 10 per condition, **p < .01)

By taking advantage of weak electrostatic charge interactions for the generation of self‐assembled nanoscale carriers, we used low molecular weight synthetic polypeptides to package IGF‐1. We tuned the ratio of the polypeptides, negatively charged pGlu and positively charged pArg, to induce self‐assembly into organized and charged nanostructures. The formation of nanoplexes was induced by the sequential addition of positively charged IGF‐1, pGlu, and pArg (Figure 1a, b). The ratios of the components were varied across a broad range to explore the size and zeta potential of the nanoplex using dynamic light scattering (DLS) coupled with zeta potential analysis. Over the molar ratios that we explored, the number averaged hydrodynamic size and zeta potential of the resulting nanoplexes as measured by DLS ranged from 100 to 850 nm and 0 to +140 mV, respectively (Figure 1c). The polydispersity index ranged from 0.1 to 0.25. To maximize penetration into the cartilage to form IGF‐1 depots, we selected the nanoplex formulation with the lowest apparent hydrodynamic radius based on DLS, which consisted of ∼10% IGF‐1 by mass. We used cryo‐electron microscopy to more directly measure the nanoplex particle size and shape. (Figures 1d and S1). Interestingly, cryo‐EM suggested a substantially smaller size (16.6 +/− 5.4 nm as determined using image analysis) for the nanoplex compared with DLS. The cryo‐EM images also clearly indicate the presence of aggregates within solution that consist of smaller nanoplexes (Figure 1d, red arrow). In the DLS measurements, there are two distinct populations of particle size. However, the cumulant method used for calculating the particle size applies greater weight to the larger scatterers. We thus conclude that the nanoparticle formulation consists predominantly of positively charged nanoplexes containing IGF‐1 that are less than 20 nm in size, as well as some larger colloidal aggregates that are comprised of these smaller nanoplexes.

**Figure 2:**
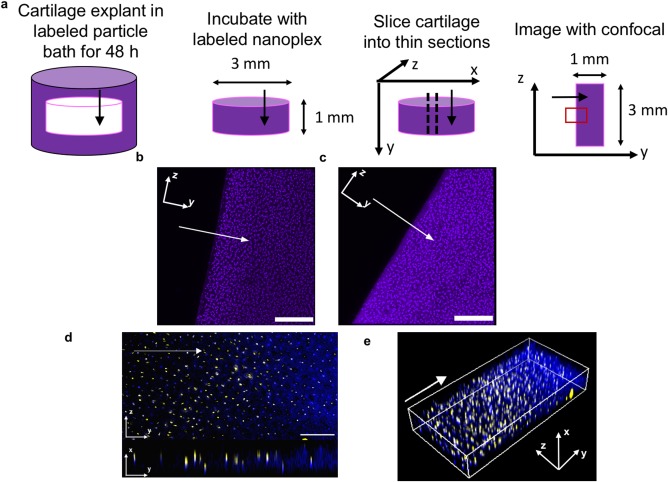
Nanoplex penetration into bovine cartilage explants. Arrows indicate the direction of diffusion. Cartilage culture media consisted of DMEM with added FBS, non‐essential amino acids, sodium pyruvate, HEPES, ascorbate, and proline unless otherwise stated. Particle treatment occurred in PBS. (a) Schematic for visualizing diffusion gradients of fluorescently labeled IGF‐1 (purple) within cartilage tissue. The red box indicates field of view shown. (b) Nanoplex delivered IGF‐1 and (c) IGF‐1 alone distributed throughout the tissue (Scale bar = 200 µm) (d) Penetration of nanoplex components (yellow: polyArg, blue: IGF‐1) throughout inflamed cartilage tissue. Inflammation in cartilage tissue was simulated by addition of 10 ng/ml of IL‐1α to cartilage media. Top: yz view, bottom: yx view. Scale bar = 100 µm (e) 3D visualization of panel (d)

We assessed the ability of the nanoplex IGF‐1 to interact with and induce proliferation in an immortalized chondrocyte cell line (CHON‐001). After 1 hr of incubation with the cell line, the IGF‐1 and polyelectrolytes appeared as punctate structures, associated with the cell membrane (Figure 1e). Furthermore, nanoplex IGF‐1 induced proliferation in a dose dependent manner, at levels comparable to free IGF‐1 at the same dose, suggesting that the general bioactivity of IGF‐1 was retained in the nanoplex (Figure 1f) (ANOVA with a Tukey post hoc test; *n* = 6 per group per time point). To confirm specific bioactivity of IGF‐1 in increasing cartilage matrix production, the sulfated glycosaminoglycan (sGAG) synthesis within ex vivo cartilage disks from a bovine joint was examined. Nanoplex and free IGF‐1 treated cartilage showed similar increases in sGAG synthesis relative to untreated control cartilage (Figure 1g) (ANOVA with a Tukey post hoc test; *n* = 10 per group). To compare the transport performance of nanoplexes versus IGF‐1 alone in the dense cartilage matrix, we incubated fluorescently labeled nanoplexes for 48 hr with ex vivo bovine cartilage disks from a bovine joint and visualized the diffusion gradients of nanoplexes and fluorescently labeled IGF‐1 in tissue sections using confocal microscopy (Figure 2a). The nanoplex delivered IGF‐1 and free IGF‐1 had a similar distribution profile throughout the section (Figure 2b, c). We then investigated transport within injured cartilage by treating the explants with IL‐1α prior to incubation with nanoplex. Both IGF‐1 and pArg were labeled to analyze nanoplex disassembly within the tissue. pArg and IGF‐1 co‐localize as the nanoplex enters the tissue, but as it moves deeper, only IGF‐1 is present (Figure 2d, e)

**Figure 3:**
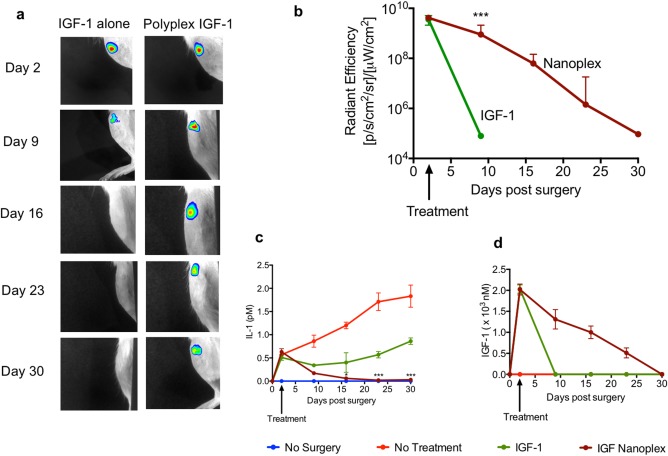
In vivo activity of IGF‐1 in a rodent OA model as a function of time. (a) Retention of IGF‐1 in a rat OA joint and (b) Quantification of IGF‐1 in the rat joint. (c) IL‐1β levels in the synovial fluid of rat OA joints and (d) Comparison of IGF‐1 retention using ELISA in the rat OA joint (*n* = 5 per condition, *p < .05, ***p < .001). Comparisons are made with the IGF‐1 group

We examined the efficacy of the nanoplex growth factor delivery system in a rat osteoarthritis model (Figure 3). We treated the animals via intra‐articular injection of the nanoplexes at 48 hr after inducing surgical destabilization of the knee joint. In live animals, we tracked the bulk retention of free and nanoplex packaged IGF‐1 by tagging the IGF‐1 component using a near‐IR fluorescent reporter that was detectable using an IVIS (Figure 3a). Free IGF‐1 was rapidly cleared within a few days after administration. In contrast, when packaged in the nanoplex, the IGF‐1 was detectable in the joint for about 4 weeks post‐injection and was 4–5 orders of magnitude higher in concentration than free IGF‐1 at day 9 (Figure 3b) (*p* < .001, Student's *t* test; *n* = 5). To monitor the level of joint inflammation, we measured IL‐1β levels in the synovial fluid of the injured joint and the contralateral joint, in which surgery was not performed. In untreated animals, IL‐1β levels increased steadily over the course of the study (Figure 3c). When free IGF‐1 was administered, there was an initial decrease in the IL‐1β concentration in the synovial fluid, which gradually increased 2 weeks after IGF‐1 injection. When treated with the nanoplex, the IL‐1β production in the joint was suppressed at a low level, comparable to the uninjured joint, over the duration of the study. We also measured IGF‐1 concentrations in the synovial fluid (Figure 3d). We observed the same trend as that of the IVIS fluorescence measurements. IGF‐1 was detectable for several weeks longer in animals treated with the nanoplex IGF‐1 compared with free IGF‐1, which was undetectable after day 9 (ANOVA with a Tukey post hoc test; *n* = 5 per group per time point for IL‐1β and IGF‐1 measurements).

**Figure 4:**
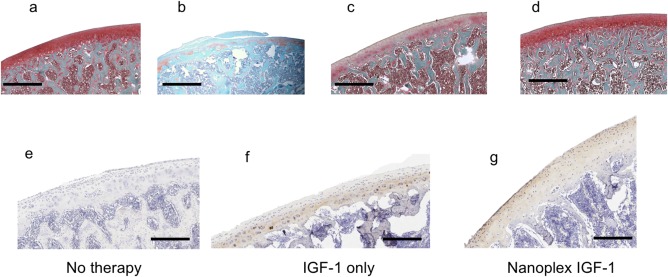
Representative histology of rat OA joints at 4 weeks. (a) Normal, uninjured joint (b) OA joint without treatment. (c) OA joint treated with 100 ng IGF‐1 alone (d) OA joint treated with IGF‐1 nanoplex (100 ng IGF‐1). Immunohistochemistry on OA joints (e) OA joint without treatment, (f) OA joint treated with 100 ng IGF‐1 alone, (g) OA joint treated with IGF‐1 nanoplex (containing 100 ng IGF‐1) (Brown: aggrecan, Purple: collagen). Scale bar: 400 μm (a–d) and 200 μm (e–g). (*n* = 5 per experimental group). The whole joint images are provided in supplementary figures S2–S8, with the magnified areas highlighted

To examine structural and morphological changes in the cartilage, we harvested the cartilage 4 weeks after surgery and stained sagittal sections with Safranin‐O (Figure 4a–d). We observed severe proteoglycan loss in cartilage in the untreated rats (Figure 4b) (*n* = 5 per group). Notably, we detected proteoglycan loss at the deep zone of articular cartilage. The thickness of the calcified cartilage zone in these rats was greater than the contralateral healthy control joints (Figure 4a), with the tidemark moving closer to the articular surface. Proteoglycan loss and calcification of articular cartilage were attenuated in treated animals. We examined aggrecan production by chondrocytes in the deep zones of the articular cartilage using immunohistochemistry (IHC) (Figure 4e–g). A qualitative comparison indicated that more chondrocytes in the deep zone produced new aggrecan in the nanoplex treated animals, and this new aggrecan was uniformly distributed throughout the articular cartilage (Figure 4g). However, in free IGF‐1 treated animals, a loss of aggrecan in the superficial zones of the cartilage was observed (Figure 4f). In untreated animals, aggrecan was not detected in the cartilage at 4 weeks (Figure 4e).

## 4 Discussion

We examined the long‐term delivery of a therapeutic growth factor (IGF‐1) within the cartilage tissue by packaging it into an electrostatically self‐assembled nanoplex carrier. The synthetic polypeptides form a complex with IGF‐1 to form nanoscale depots with extended residence time in the joint. These long‐lived depots are present in the cartilage tissue and release IGF‐1 into the synovial fluid and cartilage over a therapeutically relevant timescale for a growth factor, offering the benefit of sustained, long‐term therapy. We selected poly(glutamic acid) as the polyanion, which is a water‐soluble, biodegradable, and edible biopolymer. It is clinically used in FDA‐approved polymer‐drug conjugate systems.^16^ When used to deliver IGF‐1, we observed stable binding of the poly(glutamic acid) and the IGF‐1. Poly(arginine) has been widely examined in drug and gene delivery applications for its ability to transport molecules across cell membranes.^17^, ^18^ In our work, poly(arginine) packaged the poly(glutamic acid)‐IGF‐1 complex and conferred a net positive charge to the complex for transport through negatively charged cartilage ECM. The assembled nanoplex demonstrated co‐localization with the membrane of chondrocytes (Figure 1e), which could be due to either nonspecific charge interaction with the membrane itself or specific interaction with the extracellular IGF‐1 receptor. Electrostatic interactions of the nanoplex with the highly negatively charged cartilage matrix are anticipated to facilitate early penetration of the complex. These same interactions may also enable unpackaging of the complex in cartilage tissue. Both polypeptides used can be broken down over longer timeframes into their constituent amino acids and efficiently processed and cleared by the body.

While numerous growth factors stimulate chondrocytes to synthesize cartilage ECM,^19^ IGF‐1 is the main pro‐anabolic growth factor for articular cartilage and is crucial in maintaining cartilage homeostasis and remodeling cartilage.^20^ The role of IGF‐1 in articular cartilage metabolism has been extensively investigated in both healthy and diseased joints. In our own work, we have demonstrated in vitro that IGF‐1 stimulates proteoglycan production in a dose‐dependent manner.^21^ IGF‐1 has been demonstrated to induce the production of hyaline‐like cartilage with native mechanical properties, as opposed to fibrocartilage.^22^

The negatively charged GAG chains of aggrecan play a unique role in determining the transport properties of the cartilage.^23^ It has been estimated that the effective mesh size of these networks is on the 10 s of nanometers scale in healthy cartilage.^24^ This matrix is highly anionic and increasingly impermeable to molecules much greater than the size of albumin (∼67 kDa), depending upon their charge and conformation.^25^ The nanoplex construct, with a net positive charge and 10–40 nm size range for non‐aggregated particles, is suitable for diffusion into and through this matrix; cartilage injury may enable diffusion of even larger molecules.^26^ It is particularly notable that these nanoplex particles are soft, dynamic, charged assemblies capable of engaging with the cartilage based on electrostatics and undergoing changes in size, shape, and colloidal stability during diffusion.

To reduce the risk of infection and joint damage, intra‐articular injections are administered infrequently, with intervals that can span weeks to months.^27^ Hence, sustained delivery of the growth factor is key to the success of this strategy. Various intra‐articular drug carriers based on hydrogels, liposomes, and polymeric micro and nanoparticles have been reported.^28^, ^29^, ^30^, ^31^, ^32^ Recent in vivo studies with small nanoparticles have indicated the possibility of penetration into ∼50 micron‐thick mouse cartilage, with a multi‐day residence time within the cartilage space.^33^ Whereas many of these systems have demonstrated some level of efficacy, their applicability is limited to delivering small molecules and by long‐term instability. Only recently, have delivery systems begun to harness charge interactions, which play a critical role in mediating transport through cartilage.^34^, ^35^ Electrostatic interactions between soluble IGF‐1 and GAGs regulate transport and binding within cartilage. IGF‐1 is a basic protein (pI ∼8.5) that partitions into cartilage on the basis of size and charge; in addition, IGF‐1 binds to specific domains (IGF binding proteins) that are contained within the ECM.^36^ The advantage of a nanoplex system is that it can transport the IGF‐1 out of the high clearance rate joint space into the cartilage matrix to create depots that sustain IGF‐1 release and signaling. The nanoplex has a high cargo capacity and densely packs the IGF‐1, which comprises approximately 10% of the total mass of the particle and transports it through the cartilage tissue. Conventional bulk polymer delivery systems typically comprise about 1% of growth factor by mass, a fraction of which is bioavailable.^37^ Importantly, we observed that the IGF‐1 maintained its bioactivity in the nanoplex (Figure 1e–g). In bovine cartilage explants, we observed that a single dose of the nanoplex was sufficient to induce comparable levels of GAG synthesis as free IGF‐1. We note that the dose of IGF‐1 used in these studies was higher than what has generally been observed to saturate the chondrocyte response in vitro, and future studies will investigate dose response to IGF‐1 delivery systems. The nanoplex particles were capable of diffusing and penetrating into bovine cartilage explants within 2 days. There are a few possibilities for the mechanism of nanoplex penetration into cartilage and release of IGF‐1. Larger (> 50 nm) nanoplex aggregates may be unable to penetrate the cartilage matrix; however, these polyelectrolyte complexes are highly hydrated, soft colloidal coacervates. They may gradually break down into the observed 10–40 nm fragments and/or undergo a mode of disassembly upon interacting with negatively charged aggrecan in cartilage, thus enabling deep penetration of nanoplexes and release of IGF‐1 that can penetrate even deeper into the tissue.

While efficient transport through cartilage is desirable, overcoming joint clearance mechanisms in vivo is also crucial to transport therapeutically relevant levels of IGF‐1 into the cartilage at therapeutic timescales. Efficient lymphatic drainage rapidly removes materials from the synovial fluid resulting in an intra‐articular dwell time of proteins on the order of several hours.^38^ In inflamed joints, fluid flow to the lymphatics is enhanced resulting in even faster clearance of macromolecules.^39^ Using fluorescence and ELISA measurements, we observed that when the IGF‐1 was packaged in the nanoplex, its half‐life in the joint significantly increased as measured by IVIS fluorescence. The IGF‐1 clearance rate from the synovial fluid was constant (0.7 nM/day, *R* ^2^ = .99) confirming that lymphatic clearance was not affected by the size or amount of the therapeutic.

The breakdown of the ECM in cartilage is a hallmark of established and end‐stage OA. Cartilage matrix consists largely of collagen fibrils, key proteoglycans such as aggrecan which are bound to hyaluronan, and dozens of additional ECM molecules. This breakdown of this matrix is orchestrated in part by a range of inflammatory cytokines, including IL‐1β, that are upregulated by cells in many joint tissues in response to traumatic joint injury. Over the course of treatment following injury in the rat model, we observed that the IGF‐1 delivered from the nanoplex was able to moderate acute inflammation as measured by lowered IL‐1β concentration in synovial fluid. Free IGF‐1 lowered IL‐1β concentration for about 2 weeks post‐injection and coincided with the clearance of the free IGF‐1 from the joint. The observation of the magnitude of IL‐1β in the osteoarthritic rat joint is consistent with that observed in human osteoarthritic joints.^40^, ^41^ We measured the IL‐1β concentration in the synovial fluid, which would be sampled continuously. We note that the concentrations in harvested whole knee joints and blood maybe higher in similar OA models.^42^, ^43^ Our prior work with human cartilage and that of others has indicated that an IGF‐1‐mediated anabolic effect is the primary mechanism by which new matrix is synthesized.^15^, ^19^ However, it has also been demonstrated that IGF‐1 can suppress IL‐1β‐induced NF‐κB activation via inhibition of IκB‐α kinase, which is an anti‐catabolic mechanism.^44^ The reduction in IL‐1β levels in the synovial fluid are likely an indirect effect of suppressing inflammation.

In our prior work, we have observed quantifiable GAG loss as early as 48 hr after incubation of IL‐1α with bovine cartilage plugs,^15^, ^45^ mainly from the superficial zone. Based on this understanding of the progression of OA, we administered the therapy within 48 hr after surgery, during the acute inflammation phase, to mimic an early‐intervention treatment. It is unlikely that there were any major macroscopic defects in the cartilage structure at this timepoint post‐surgery, though tissue GAG loss has likely been initiated. The rat joint injury model used in the study has been well characterized, in which minor focal defects appear at around 1 week after injury and worsen into serious defects by 4 weeks.^45^ In this study, the cartilage maintained its structure when IGF‐1 was delivered from the nanoplex and was morphologically similar to uninjured cartilage. Safranin‐O staining indicated that the cartilage tidemark was well defined, with an even distribution of proteoglycans. We observed greater loss of proteoglycans when only free IGF‐1 was used. The untreated control resulted in near‐complete loss of proteoglycan, with the tidemark moving close to the articulating surface. IHC indicated qualitatively that aggrecan content after ACLT with nanoplex treatment was more similar to that in the normal uninjured joint. Taken together, the observations suggest our treatment likely mitigates degeneration to some extent and induces new tissue formation to repair the tissue loss that could not be prevented over the time frame of the study.

The materials used in this study are readily translatable to clinical application. Both glutamic acid and arginine are on the FDA inactive excipient list. Recombinant human IGF‐1 is a clinically used protein therapeutic for the treatment of growth failure and growth factor deficiency disorders and is manufactured for these indications.^46^ In the nanoplex system, all materials are held together by electrostatic charge, and used without chemical or biological modification. All of these factors ease the clinical translation of this approach. In the standard rodent model of joint injury described in the study, we demonstrated that the nanoplex system delayed cartilage degradation in a mechanically destabilized joint complex. Our studies demonstrated the efficacy of the nanoplex system in preventing cartilage degradation over a period of 4 weeks post‐injury. Long‐term cartilage degradation is inevitable in the surgical model of mechanical joint destabilization in a rodent. We envision that the nanoplex system will supplement the standard pharmacological therapy that is administered after a significant joint injury, such as an ACL tear, which is well correlated with post‐traumatic OA. Prior to a human clinical trial, we anticipate that the nanoplex system will need to be tested over a longer timescale in a larger animal OA model (e.g., sheep) in which the injury and intervention more closely mimics that of a human joint injury. These larger animal trials will also help determine the nanoplex dose range for therapeutic benefit in humans. In clinical trials, both short‐term (alleviating inflammation) and long‐term (preventing/delaying onset of OA) patient outcomes and data will help guide decisions for clinical use.

## 5 Conclusions

Regenerative strategies to replace damaged cartilage are often used, but only after the clinical symptoms manifest, at which time it is often too late to rescue degraded cartilage tissue. This work demonstrates a strategy to potentially prevent cartilage degradation through the use of a charged carrier system to transport an anabolic protein into the cartilage for therapeutic efficacy. The nanoplex offers a platform for combining multiple complementary and synergistic growth factors to promote cartilage repair and a way to efficiently deliver potent biologics through the dense cartilage ECM barrier. The work highlights a potential interventional therapy for trauma‐induced joint injuries that can potentially reverse cartilage damage and prevent progression to irreversible joint degradation.

## Author contributions

N.J.S., B.C.G., M.A.Q. and P.T.H. designed the experiments. N.J.S., B.C.G., M.A.Q. and M.N.H. conducted the experiments and analyzed the data. Y.K. and A.J.G. assisted in analyzing data. All authors assisted in preparing the manuscript and approved the final version. The authors have no associated competing interests.

## Supporting information

Additional Supporting Information may be found online in the supporting information tab for this article.

**FIGURE S1** Particle size analysis from cryo‐electron microscopy. (a) Representative image in grayscale. (b) Image thresholded into black and white for particle analysis. (c) Particles identified by particle analysis algorithm using ImageJ. (d) Histogram of particle sizes

**FIGURE S2** Whole knee image of uninjured joint corresponding to Fig. 4a

**FIGURE S3** Whole knee image of untreated joint corresponding to Fig. 4b

**FIGURE S4** Whole knee image of IGF‐1 only treated joint corresponding to Fig. 4c

**FIGURE S5** Whole knee image of IGF‐1 nanoplex treated joint corresponding to Fig. 4d

**FIGURE S6** Whole knee image of untreated joint corresponding to Fig. 4e

**FIGURE S7** Whole knee image of IGF‐1 nanoplex treated joint corresponding to Fig. 4f

**FIGURE S8** Whole knee image of IGF‐1 nanoplex treated joint corresponding to Fig. 4g
