# Supplementary figures and images for: Synthetic nanoscale electrostatic particles as growth factor carriers for cartilage repair
Source: Bioeng Transl Med. 2016 Nov 18;1(3):347–56. doi: 10.1002/btm2.10043 (PMC5457159; doi:10.1002/btm2.10043)

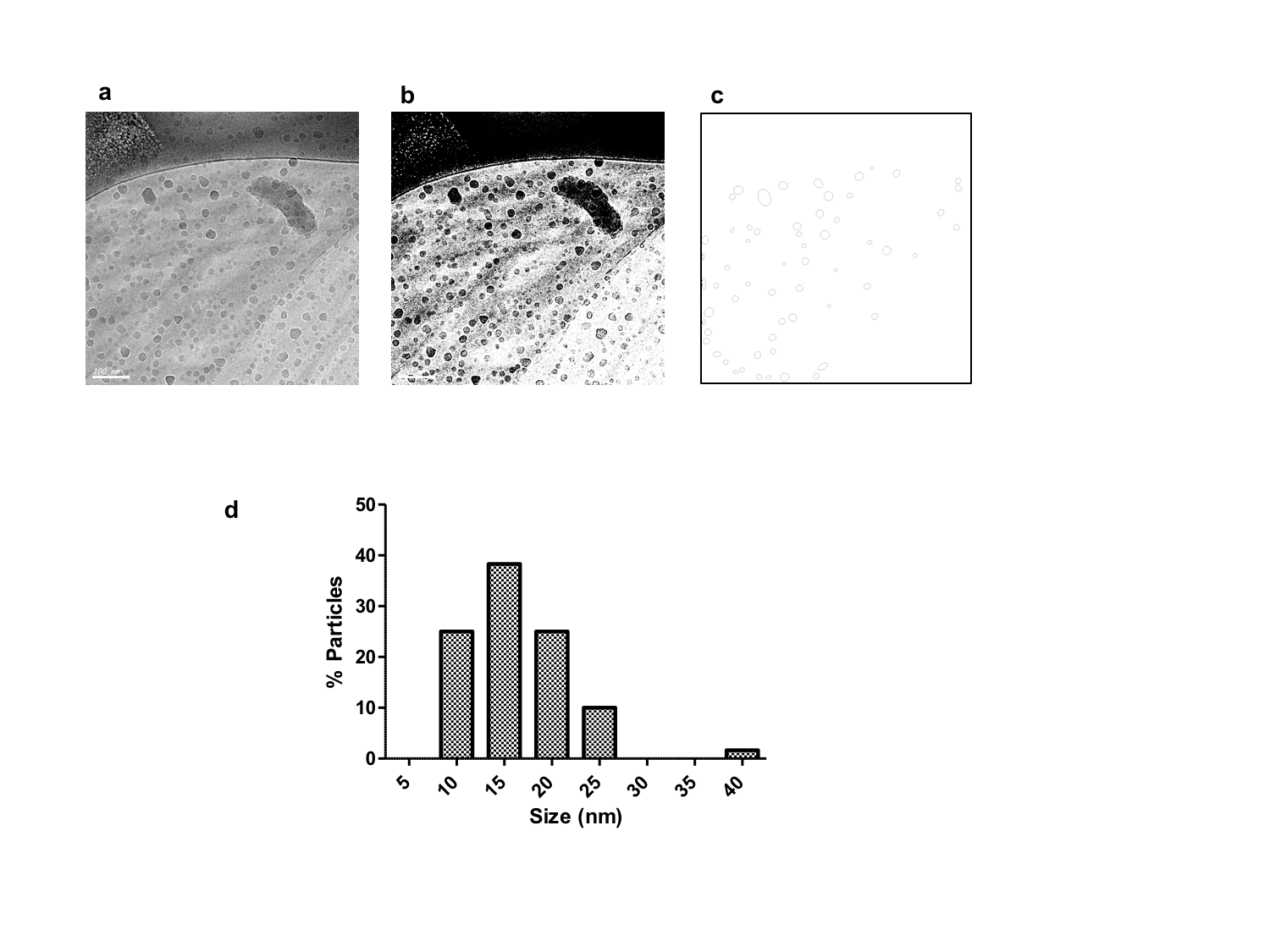

Supplement: Supplementary file 1 — FIGURE S1 Particle size analysis from cryo‐electron microscopy. (a) Representative image in grayscale. (b) Image thresholded into black and white for particle analysis. (c) Particles identified by particle analysis algorithm using ImageJ. (d) Histogram of particle sizes [file BTM2-1-347-s001.tiff]

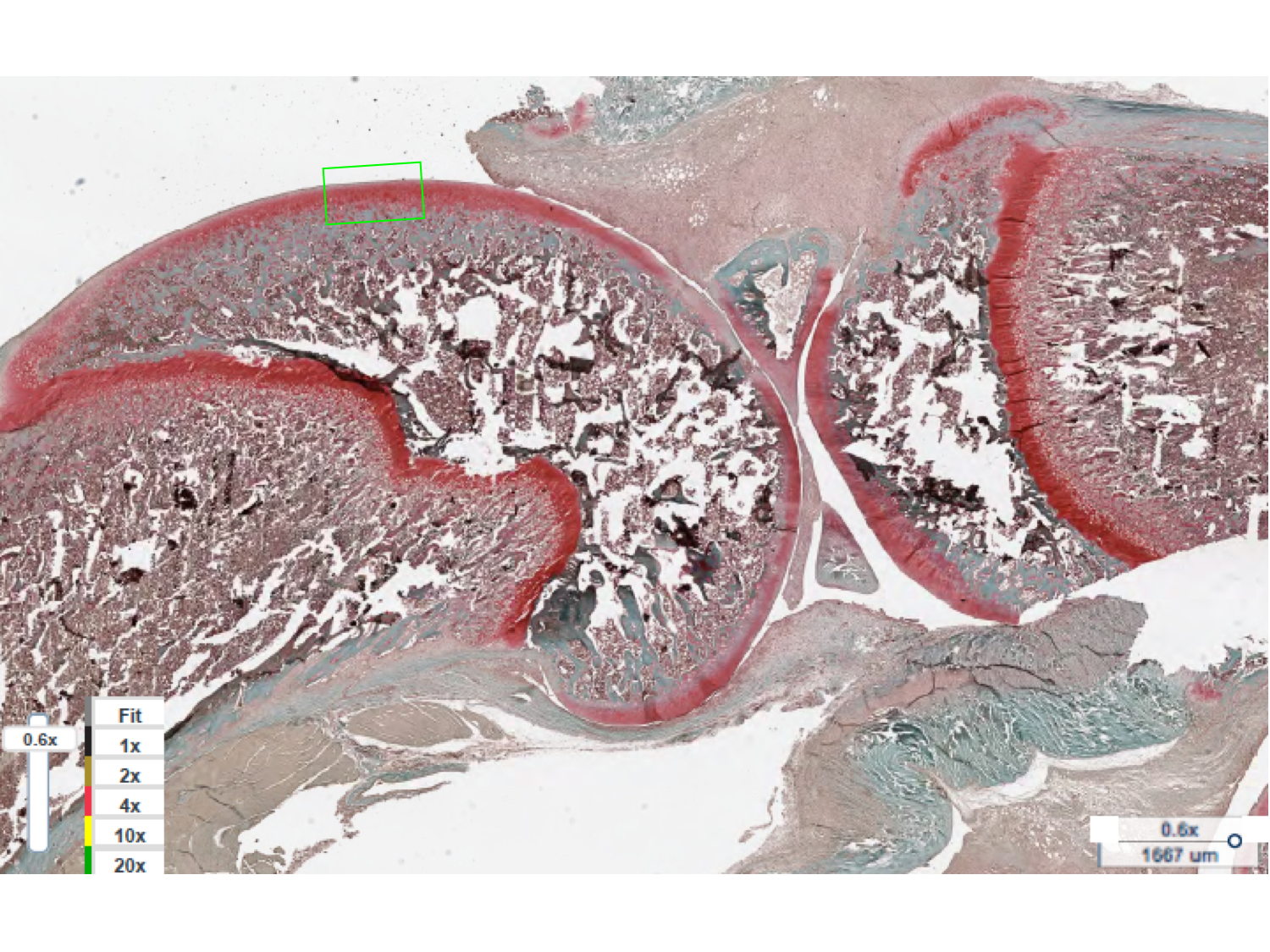

Supplement: Supplementary file 2 — FIGURE S2 Whole knee image of uninjured joint corresponding to Fig. 4a [file BTM2-1-347-s002.tiff]

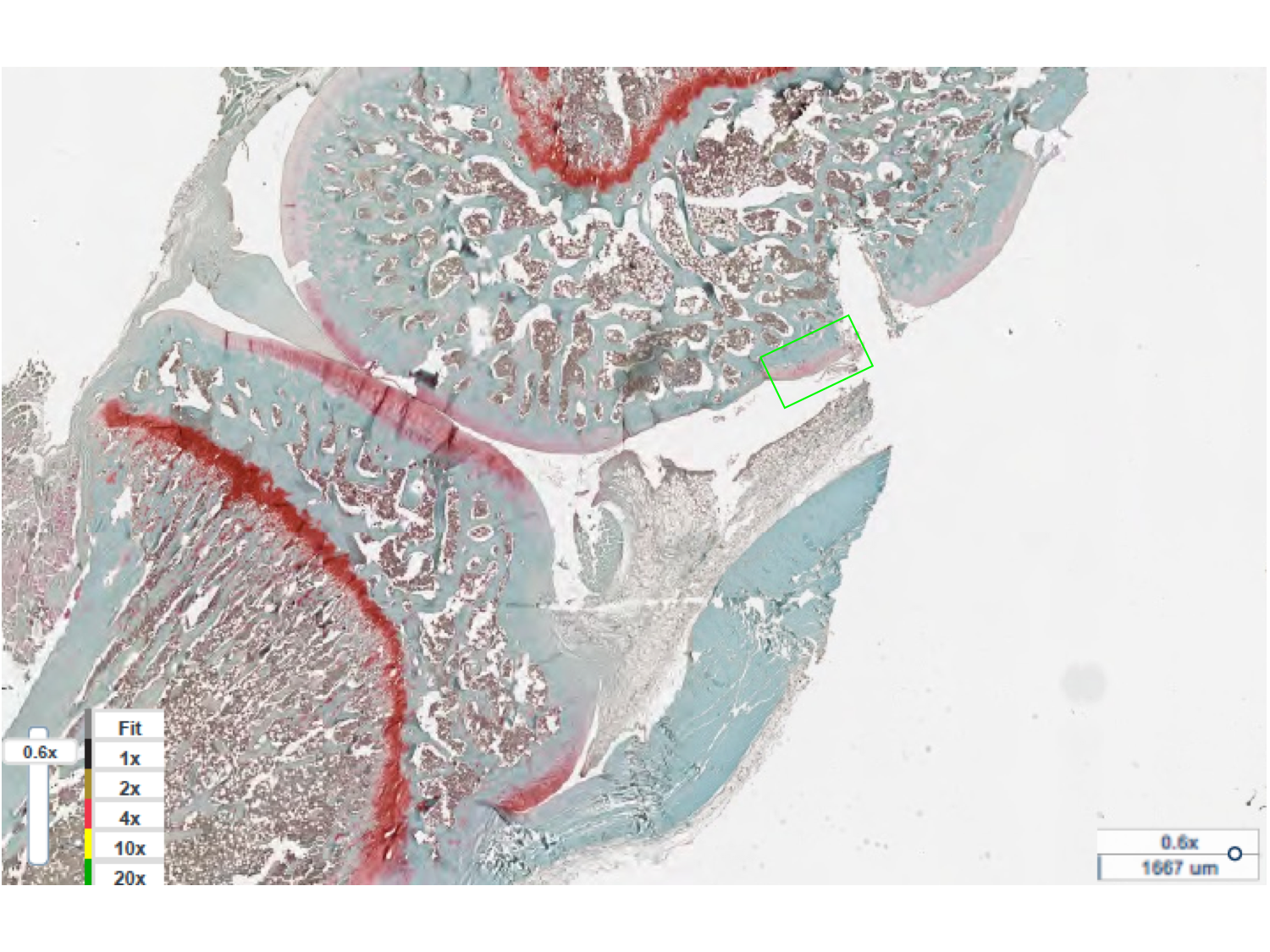

Supplement: Supplementary file 3 — FIGURE S3 Whole knee image of untreated joint corresponding to Fig. 4b [file BTM2-1-347-s003.tiff]

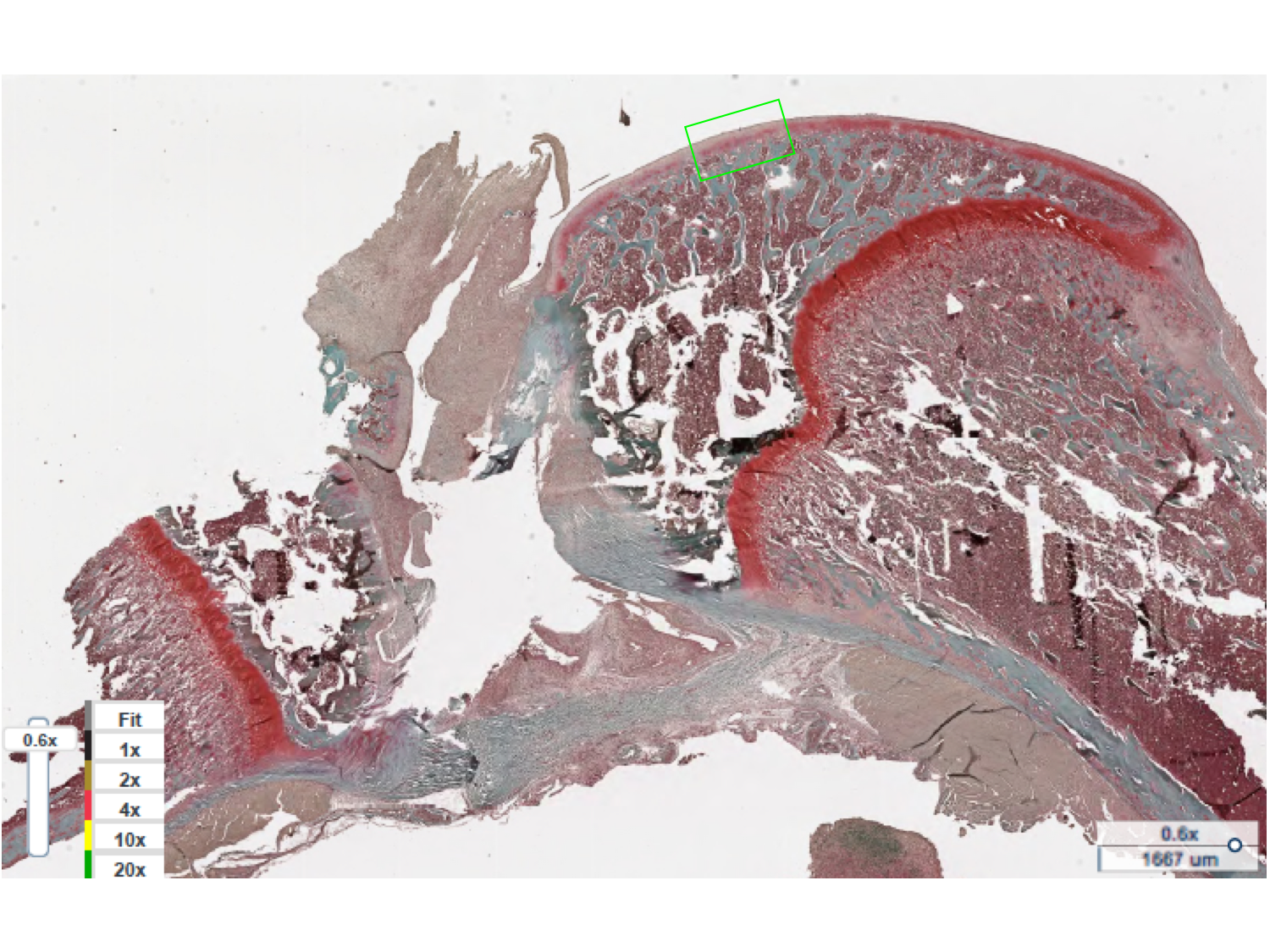

Supplement: Supplementary file 4 — FIGURE S4 Whole knee image of IGF‐1 only treated joint corresponding to Fig. 4c [file BTM2-1-347-s004.tiff]

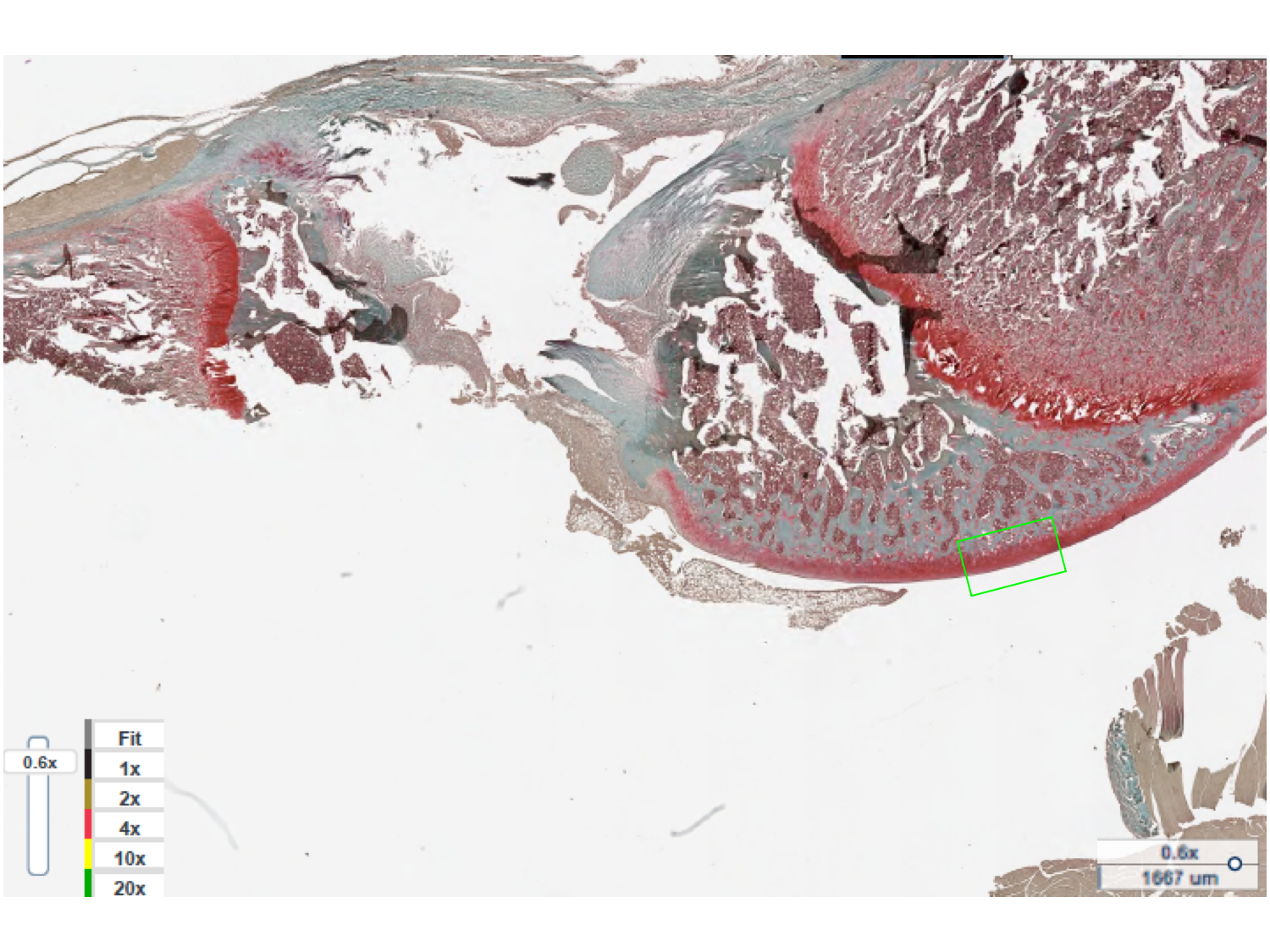

Supplement: Supplementary file 5 — FIGURE S5 Whole knee image of IGF‐1 nanoplex treated joint corresponding to Fig. 4d [file BTM2-1-347-s005.tiff]

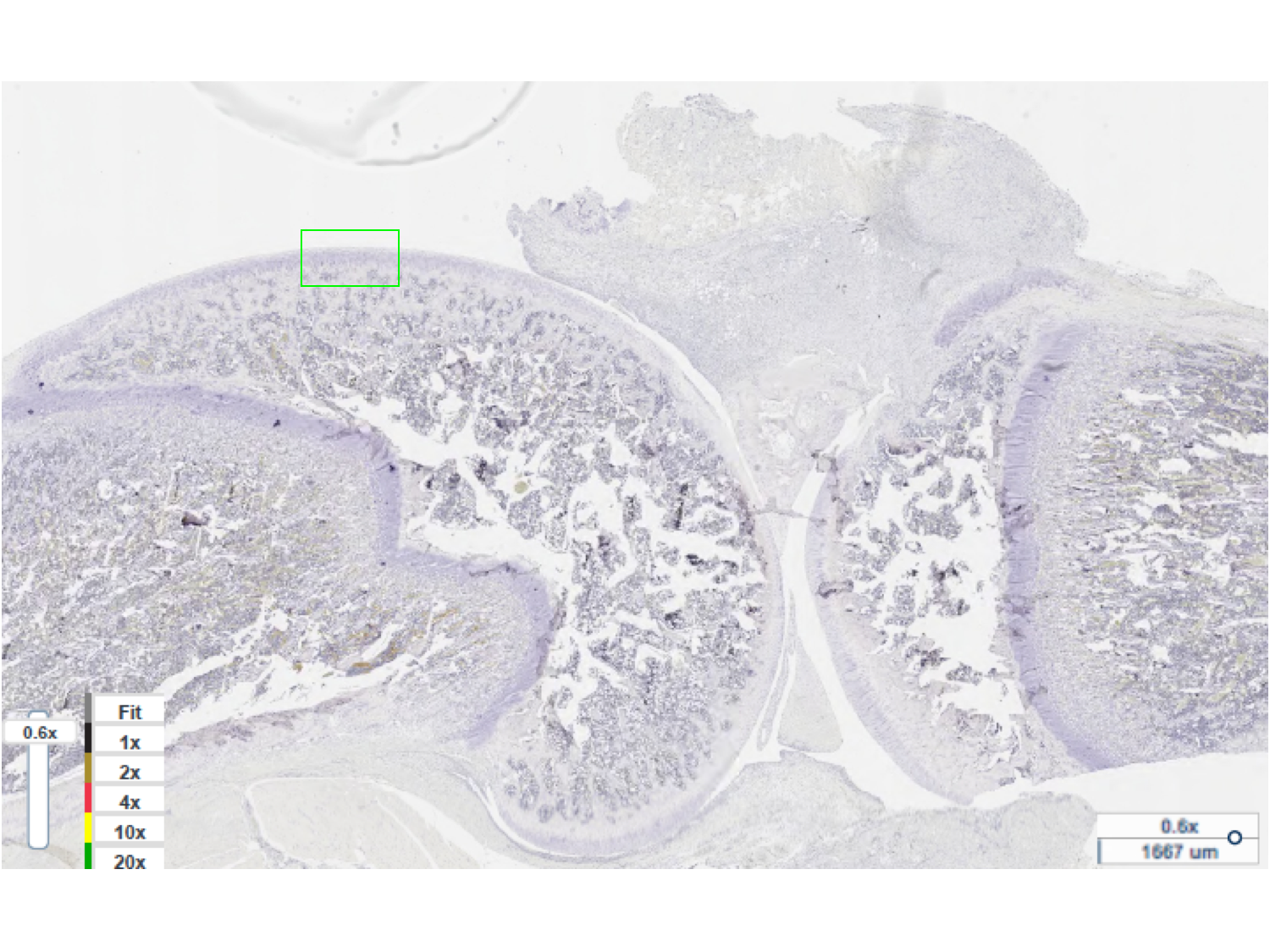

Supplement: Supplementary file 6 — FIGURE S6 Whole knee image of untreated joint corresponding to Fig. 4e [file BTM2-1-347-s006.tiff]

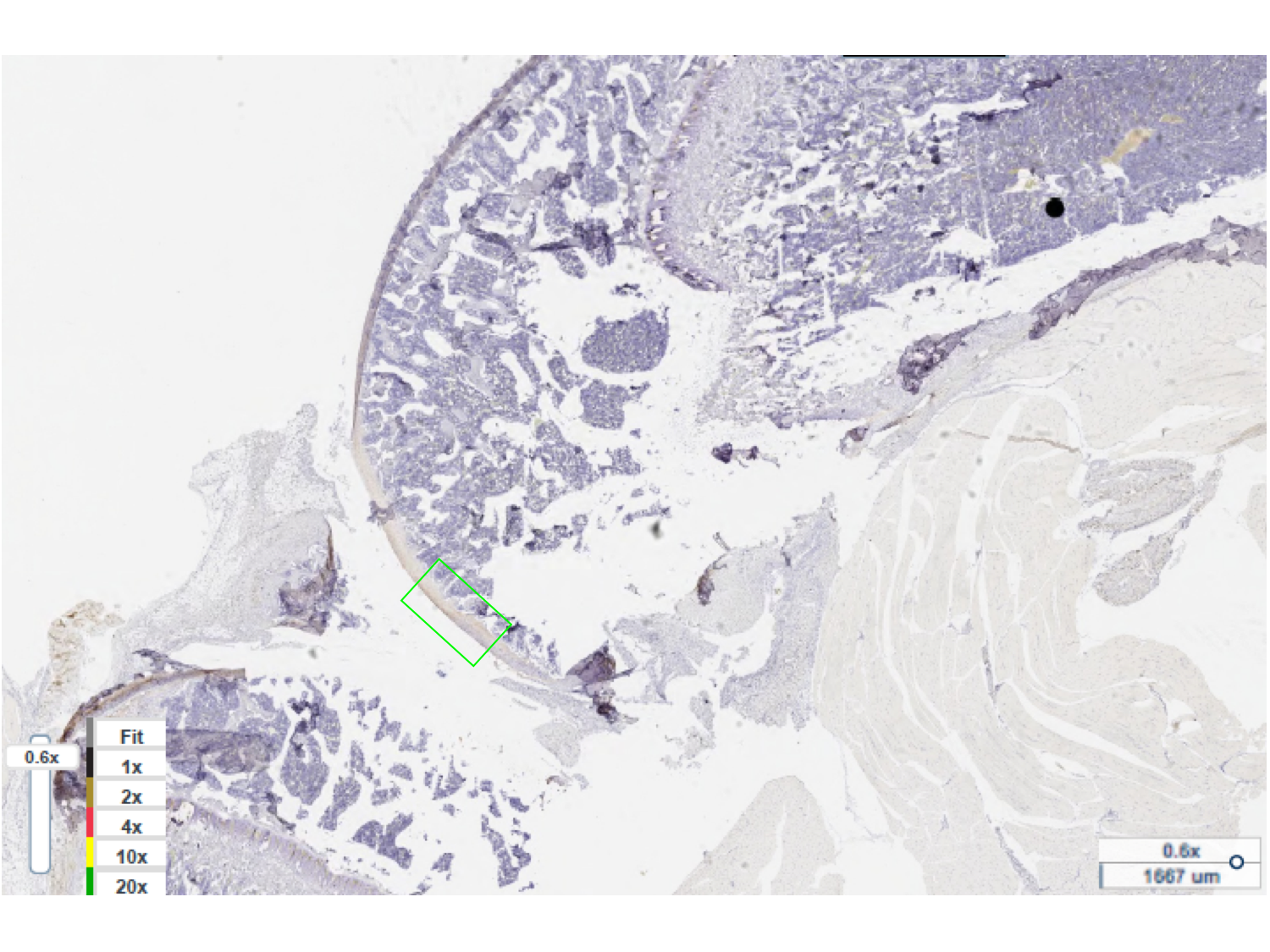

Supplement: Supplementary file 7 — FIGURE S7 Whole knee image of IGF‐1 nanoplex treated joint corresponding to Fig. 4f [file BTM2-1-347-s007.tiff]

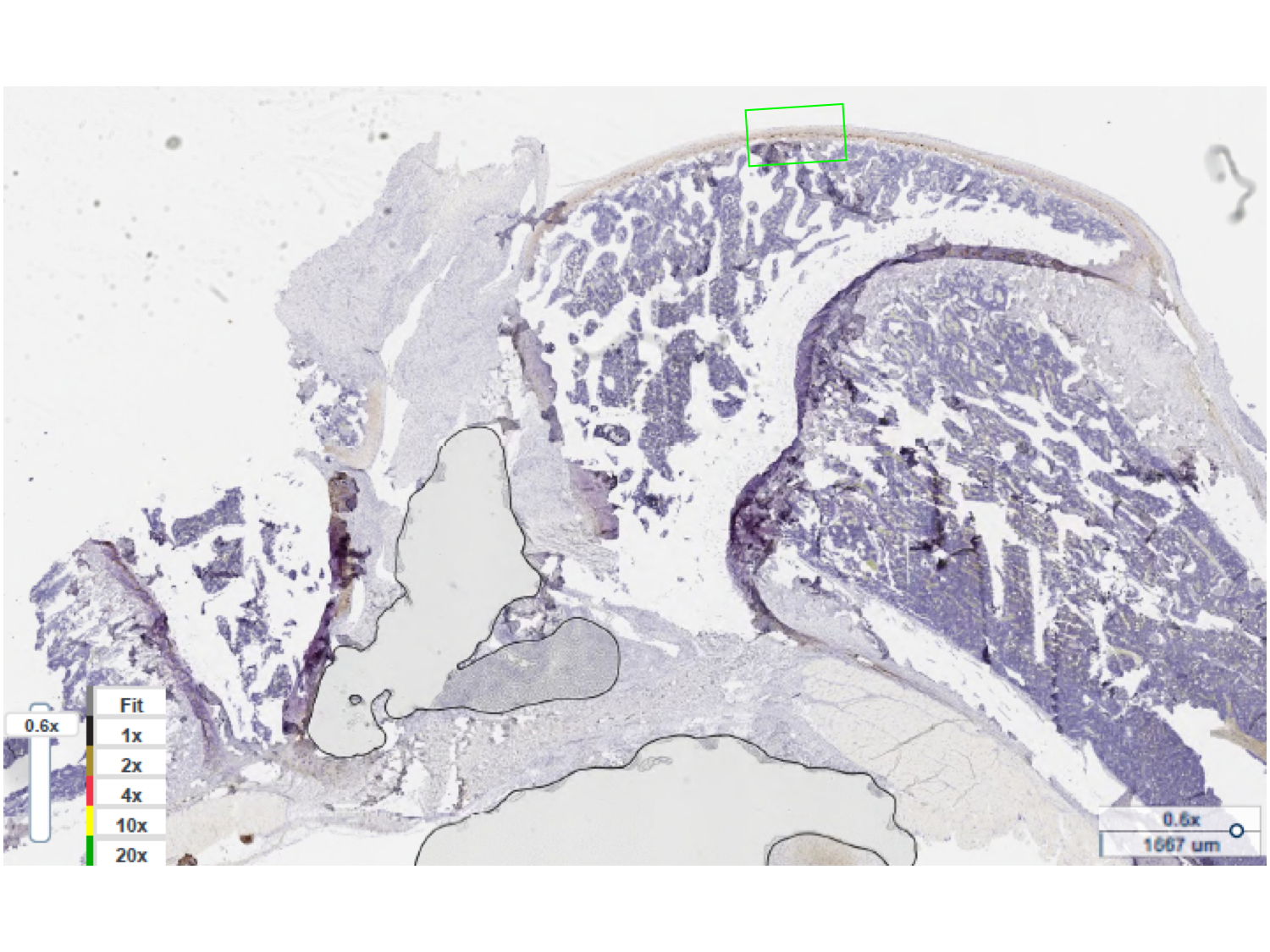

Supplement: Supplementary file 8 — FIGURE S8 Whole knee image of IGF‐1 nanoplex treated joint corresponding to Fig. 4g [file BTM2-1-347-s008.tiff]
